# Supplementary material for: Developmental Screening Tools Used with First Nations Populations: A Systematic Review
Source: Int J Environ Res Public Health. 2022 Nov 24;19(23):15627. doi: 10.3390/ijerph192315627 (PMC9739511; doi:10.3390/ijerph192315627)
Supplement: Supplementary file 1 [file ijerph-19-15627-s001.zip › ijerph-2003827-supplementary.pdf]

Supplementary Tables

Supplementary Table S1

Search strategy and search terms

Database	Date	Search
**PsychInfo**		(noft("child" OR "infant*" OR "baby" OR "preschool") AND noft("milestone*" AND "surveillance" OR ("screening tool" OR "screening tools") OR "screening measure*" OR "screening assessment*")) AND noft("first nations" OR "indigenous" OR "Aboriginal" OR "native" OR "Torres Strait Islander*")
**Embase**		1 child/ 2214799<br>2 infant.mp. [mp=title, abstract, heading word, drug trade name, original title, device manufacturer, drug manufacturer, device trade name, keyword heading word, floating subheading word, candidate term word] 923253<br>3 baby.mp. [mp=title, abstract, heading word, drug trade name, original title, device manufacturer, drug manufacturer, device trade name, keyword heading word, floating subheading word, candidate term word] 83245<br>4 preschool.mp. [mp=title, abstract, heading word, drug trade name, original title, device manufacturer, drug manufacturer, device trade name, keyword heading word, floating subheading word, candidate term word] 684845<br>5 1 or 2 or 3 or 42897745<br>6 milestone.mp. [mp=title, abstract, heading word, drug trade name, original title, device manufacturer, drug manufacturer, device trade name, keyword heading word, floating subheading word, candidate term word] 8878<br>7 (surveillance or screening tool or screening measure or screening assessment).mp. [mp=title, abstract, heading word, drug trade name, original title, device manufacturer, drug manufacturer, device trade name, keyword heading word, floating subheading word, candidate term word] 386010<br>8 (first nations or indigenous or Aboriginal or native or Torres Strait Islander).mp. [mp=title, abstract, heading word, drug trade name, original title, device manufacturer, drug manufacturer, device trade name, keyword heading word, floating subheading word, candidate term word] 338191<br>9 5 and 6 and 7 and 8 1<br>10 limit 9 to (human and english language) 1
**PubMed**		Search: ((((("child") OR ("infant") OR ("baby") OR ("preschool") AND ("milestone") AND (surveillance) OR ("screening tool*") OR ("screening measure") OR ("screening assessment") AND ((clinicalstudy[Filter] OR clinicaltrial[Filter] OR observationalstudy[Filter] OR randomizedcontrolledtrial[Filter]) AND (humans[Filter]) AND (2014/1/1:2022/5/18[pdat]) AND (english[Filter]) AND (allinfant[Filter] OR infant[Filter] OR

		preschoolchild[Filter]))) AND (first nations)) OR (indigenous)) OR (Aboriginal)) OR (native)) OR (Torres Strait Islander) Filters: Case Reports, Clinical Study, Clinical Trial, Observational Study, Randomized Controlled Trial, Humans, English, Infant: birth-23 months, Infant: 1-23 months, Preschool Child: 2-5 years
